# RAW 264.7 macrophage cell line as a metabolomic model for analyzing and understanding inflammation processes

**DOI:** 10.1007/s00210-026-05038-w

**Published:** 2026-02-12

**Authors:** Omar Arroyo-Xochihua, Rossana C. Zepeda, Alberto Sánchez-Medina

**Affiliations:** 1 Doctorado en Ciencias Biomédicas, Centro de Investigaciones Biomédicas, Universidad Veracruzana, Xalapa, Veracruz, México; 2 Facultad de Bioanálisis, Universidad Veracruzana, Xalapa, México; 3 Laboratorio de Biomedicina Integral y Salud, Centro de Investigaciones Biomédicas, Universidad Veracruzana, Xalapa, Veracruz, México; 4 Laboratorio de Farmacología y Quimiometría, Instituto de Química Aplicada, Universidad Veracruzana, Xalapa, México; 5 Laboratorio de Diagnóstico Molecular, Centro de Investigaciones Biomédicas, Universidad Veracruzana, Xalapa, Veracruz, México

**Keywords:** Metabolomics, Immune system, RAW 246.7 cell line, Biomarkers, Analytical methods

## Abstract

The functionality of cellular models to provide relevant and functional information has been demonstrated. Several stimuli can result in intracellular changes that can be tracked through omics sciences, such as metabolomics, a valuable tool for characterizing and quantifying metabolic changes associated. RAW 264.7 macrophage cell line is a polarizable immune cell, making it a classic cellular model for the analysis of inflammatory processes, diverse stimuli provide an overview of the cell-stimulus interaction. Although the procedures for conducting a metabolomic study, from the collection of treated cells to the obtention of the data generated, have not yet become widespread, most research using this cell line indicates that there are common metabolites, and their variation in the metabolic pathways could be fundamental for performance and function of the immune cell. Thus, in this review, we describe the application of RAW 264.7 macrophages as a metabolomic model, highlighting the sample collection processes, commonly used analytical methods, software for data analysis, multivariate analysis used for the characterization of metabolites and how these changes can help to understand inflammatory processes.

## Introduction

Cell culture studies are a valuable approach for characterizing the specific adsorption, distribution, metabolism, and excretion processes of stimuli, such as drugs or general chemicals, in a living organism. Data generated can complement the understanding of the associated mechanisms at genomic, transcriptomic, proteomic, and metabolomic levels (Zhao 2023). Relevant metabolic data can be monitored through various omics levels in a sequence established to categorize biochemical activities, as illustrated by the central dogma of molecular biology (Subramanian et al. 2020). Biological systems and disease progression can be studied at various omics levels such as genes, proteins, and metabolites (Gonzalez-Covarrubias et al. 2022; Muthubharathi et al. 2021). Advances in current analytical technologies, have allowed the study of biological systems at a more detailed level using data produced across multiple omics sciences, collectively referred to as multiomics data, transforming the field of biology and medicine (Hasin et al. 2017).

The metabolome is the most dynamic and sensitive indicator of a phenotype at molecular level, aiming to comprehensively map all biochemical reactions in a biological system (Ashrafian et al. 2021; Sindelar and Patti 2020). Metabolites can be analyzed using standard chemical analysis tools, including mass spectrometry (MS) and nuclear magnetic resonance (NMR) analysis. Since the 1980s, MS techniques have been combined with gas chromatography (GC) or liquid chromatography (LC), resulting in powerful coupled methods, such as GC–MS and LC–MS. Today, NMR spectroscopy, GC–MS and LC–MS are the most widely used analytical tools for metabolite profiling, often paired with multivariate analysis (Sun and Xia 2024; Misra 2021). Currently, the use of organ tissues or cell lines is becoming a source of chemical data to understand their metabolomic processes (Nalbantoglu 2019) and appears to be a promising approach for diagnosing and identifying essential metabolic features associated with specific pathological and physiological conditions. It can also help to understand how treatments work or in the search of new drugs. Metabolites (as end products) share the same fundamental chemical structure, regardless of the organism (Monteiro et al. 2013; Di Minno et al. 2022).

The systematic study of metabolite profiles could help to identify changes in response to external stimuli, such as drug treatments. Multiple cell lines have been used to examine changes in metabolic pathways, explaining how these processes are regulated through metabolite interactions (Saigusa et al. 2021). Therefore, the availability of multiple cell line collections has enabled the analysis of pathologies affecting the world’s population, understanding what occurs at intracellular level in cancer, stress, hyperglycemia, antipathogen, and anti-inflammatory processes, among others (Gonzalez-Covarrubias et al. 2022).

The immune system plays a key role in protecting the human body; immune cells are crucial in mapping, amplifying cell recruitment, and repairing damaged areas. Inflammation is considered a disruption of organ homeostasis, triggered by autoimmune, physical, and pathogenic conditions. Although inflammation involves a cascade of coordinated biochemical events, the nature of the stimulus determines and maps the changes in the metabolic pathways (Gusev and Zhuravleva 2022). Macrophages are crucial mediators that promote the recruitment of other myeloid cells through the secretion of interleukins and chemokines, activating enzymatic and phagocytic processes to control the damaged area (Meizlish et al. 2021). Macrophages are known for their adaptability and ability to polarize and differentiate into various cell types, and their phenotype varies significantly depending on the physiological conditions (Chen et al. 2021). RAW 264.7 cells were derived from a tumor caused by the Abelson murine leukemia virus in BALB/c mouse (Raschke et al. 1978). Numerous reports indicate that this model is particularly suitable for the analysis of inflammatory parameters and serves as a complementary tool.

**Fig. 1:**
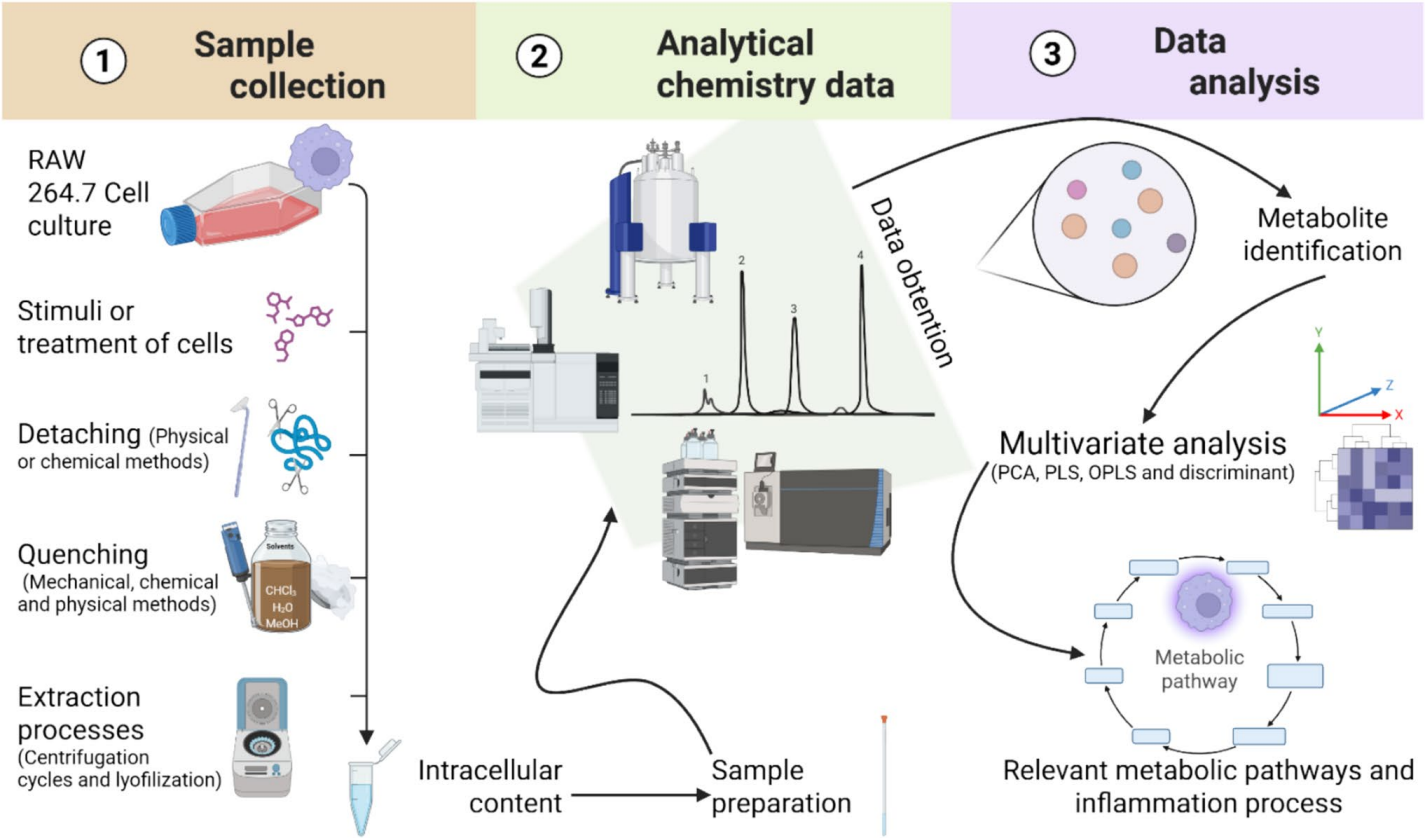
Classical steps in a metabolomic study using cell lines. Created in https://BioRender.com

Although several reports have used in-house procedures for sample collection, different analytical instruments for data obtention and the analysis is performed with different software; many of the reported metabolites in macrophage RAW 264.7 studies are involved in energy and antioxidant function or amino acid and phospholipid metabolism. Interestingly, each metabolite is also involved in macrophage functions and immunological processes. Most of these metabolites are important and individually influence specific inflammatory processes; however, Multivariate Statistical Analysis (MVA) will focus on those that have the greatest impact on the effect being evaluated. This review analyzes the use of RAW 264.7 macrophage cells as a metabolomic model to understand inflammatory processes, highlighting critical procedures, including sample collection, analytical methodologies for metabolite characterization, and the application of multivariate analysis techniques. Additionally, a comprehensive review of the role of several metabolites in inflammatory mechanisms has been carried out (Fig. 1).

## RAW 264.7 macrophage cell line as an inflammatory model

RAW 264.7 macrophage cell line has been widely used as a model for inflammation. The expression of multiple immune receptors and markers allows the emulation of an inflammatory response. They are used to test synthetic drugs, as well as chemical entities and extracts from terrestrial and marine organisms (Baek et al. 2020; Kang et al. 2022; Trinh et al. 2020; Yu et al. 2021; Fajriah et al. 2020; Bhardwaj et al. 2020).

Macrophages polarize from an M0 phenotype into M1 macrophages and can further be polarized to M2 macrophages, performing proinflammatory and anti-inflammatory functions, respectively. LPS, interferon- gamma (IFN- γ) and tumor necrosis factor (TNF) cause metabolic modifications, including alterations in amino acid, purine and energy metabolism, leading to overproduction of inflammatory cytokines such as interleukine-1β (IL-1β), interleukine-6 (IL-6) and subsequent host inflammation (Wang et al. 2024). On the other hand, M2 macrophages and their subtypes M2a, M2b, M2c and M2d are involved in the production of interleukine-4 (IL-4), interleukine-13 (IL-13), and pro-angiogenic factors such as transforming growth factor β (TGF-β) and Vascular Endothelial Growth Factor (VEGF), leading to the production of immunosuppressive interleukine-10 (IL-10). M2 macrophages are involved in the resolution of inflammatory responses by removing debris, remodeling tissue, and promoting angiogenesis (Pei and Yeo 2016; Sezginer and Unver 2024).

**Fig. 2:**
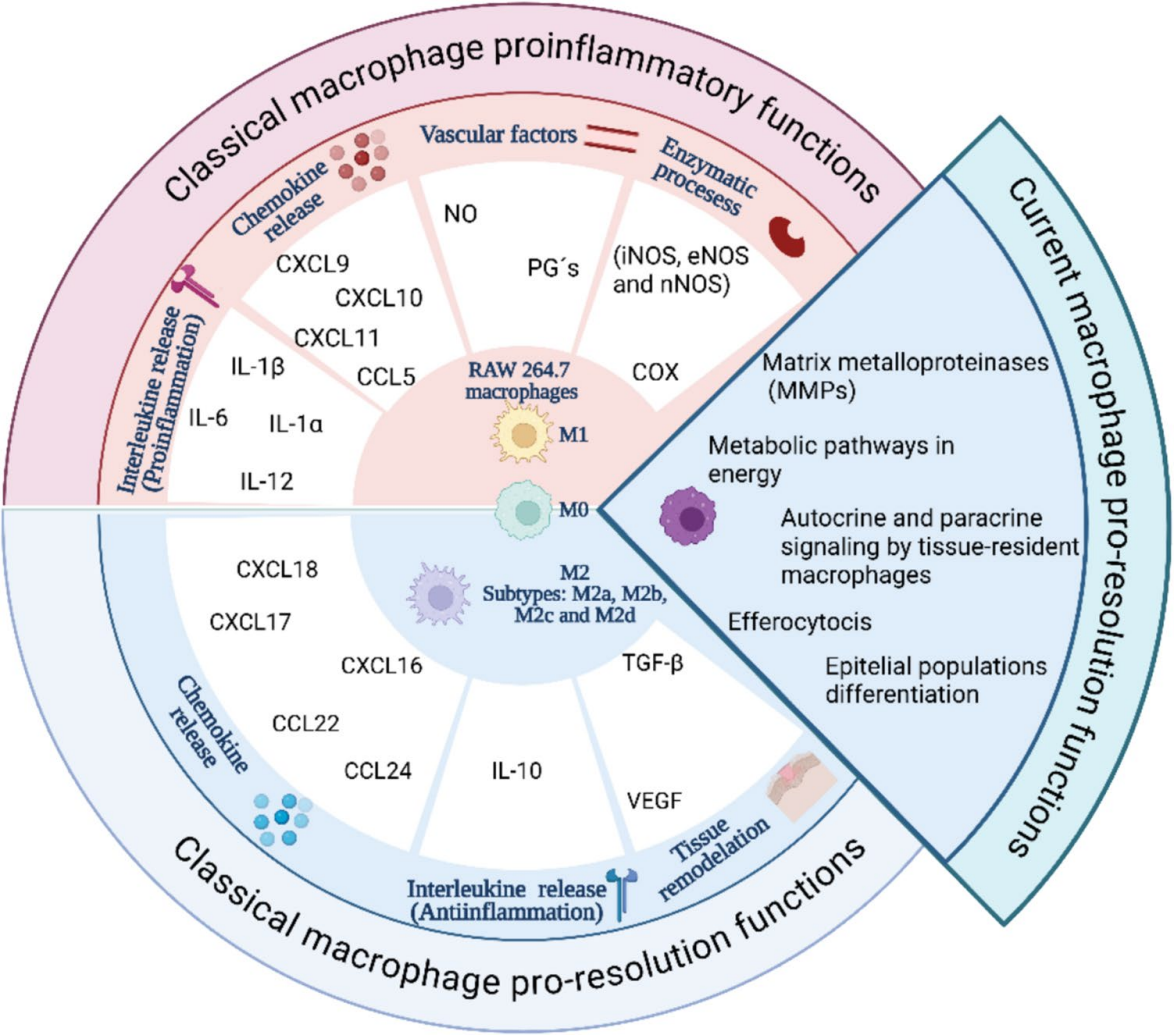
Inflammatory mechanisms in the polarization of RAW 264.7 macrophages with proinflammatory and pro-resolution functions. Adapted from (Yao et al. 2019; Rodríguez-Morales and Franklin 2023). Created in https://BioRender.com

At present, several other mechanisms have been associated with the resolution of inflammation. Macrophages release neutral extracellular proteases, such as serine proteases, cathepsins, and metalloproteinases (MMPs). MMPs are implicated in innate cell modulation by their recruitment to repair the area (Newby 2008). On the other hand, it has been shown that resident tissue macrophages communicate not only in an autocrine manner, but also in a paracrine manner, modulating the cellular and epithelial immune response in the inflamed region (Katkar and Ghost 2023). Additionally, efferocytosis is a process that removes dead and dying cells, preventing secondary necrosis and exacerbated inflammation where apoptotic cells are detected and then are bound. Finally, proinflammatory macrophages decrement in population and increase the arrival of resident and reparative macrophages. (Fig. 2) (Razi et al. 2023; Schilperoort et al. 2023).

When physical barriers of the organism are overcome, the inflammatory triggering stimulus enters and interacts with circulating or resident cells, and members of the immune system emit alerts of damaged tissue including the release of inflammasome proteins (Man and Kanneganti 2015). Macrophages non-specifically initiate their polarization, starting the synthesis of cytokines and chemokines that will allow the attraction of specialized cells to repair the area (Gordon and Martinez-Pomares 2017; Ma et al. 2019).

A proinflammatory state or disturbance in function includes five cardinal signs proposed by Paracelsus: redness, flushing, pain, edema, and loss of function, which are the result of a coordinated biochemical response and reflected in inflammasome (Serhan 2017). Cellular damage caused by microorganisms or physical, chemical and mechanical stimuli generates small molecule recognition sequences or pathogen-associated molecular patterns (PAMPs) and damage-associated patterns (DAMPs) that are recognized by pattern recognition receptors (PRRs) of cells of innate immunity (action of macrophages, mast cells, neutrophils, eosinophils and basophils), which progressively activate a series of mediators that promote increased levels of eicosanoids indicating vascular damage (Arulselvan et al. 2016). Reddening and edematization are the first physical changes in the area; afterwards, vascularization exhibits vasodilation and improved permeability. Then, an outflow of antimicrobial agents, complement fragments, platelet activators, and histamine infiltrates into the damaged area (Rossi et al. 2021).

Examples of proinflammatory mechanisms are mediated by enzymatic activity of inducible nitric oxide synthase (iNOS), with production of vascular lumen mediator nitric oxide (NO), Cyclooxygenase-1 and 2 (COX-1 and COX-2), mediators from membrane phospholipids such as prostaglandins E2, D2, I3 (PGE2, PGD2, PGI3), leukotrienes B4, C4, D4, E4 and thromboxane A2, a cellular accumulation mediated by participation of chemokines such as monocyte chemoattractant protein-1 (MCP-1), interleukins such as TNF-α that impact in nuclear translocation of the Nuclear Factor kappa B (NFκB) and the subsequent synthesis of cytokines (Juárez-Portilla et al. 2019).

A deeper understanding of how inflammation interacts with cellular metabolism could offer new insights into the treatment of inflammatory disorders. However, due to the complex nature of the interconnected metabolic pathways between various organs, tissues, and cells, a systems biology strategy, known as metabolomics, is essential to assess and understand these metabolic alterations (Fitzpatrick and Young 2013). Inflammation metabolomics simultaneously studies biomarkers that are normally obtained from various separate biochemical tests. RAW 264.7 macrophage cell line expresses several inflammatory mediators previously discussed. So, the determination of which metabolites increment or decrement will enable the understanding of their influence in the inflammatory response when a stimulus or treatment is applied (Liu et al. 2017; Liu and Locasale 2017). Thus, the following sections will detail the steps that must be carried out once the stimulus/treatment has been applied and a metabolomic study is to be performed with this cell line.

## Metabolite extraction methods

The extraction method must be standardized to ensure it is robust, reproducible, easily performable, and capable of capturing the intracellular metabolism. The success of a metabolomic study depends largely on the rapid arrest or suppression of metabolism and the extraction of metabolites in such a way that accurately represents the levels of endogenous metabolites found in the original living cell (Alseekh et al. 2021). High-quality, reliable metabolomics datasets are crucial for making correct interpretations. Methodologies for intracellular metabolomics are available; however, it is known that research teams have adopted in-house procedures (Römisch-Margl et al. 2012).

First, the number of cells dictates the overall volume of metabolites. Consequently, any change in the count of harvested cells may lead to deceptive variations in cell. This relies on the density of seeding cells, duration of culture or treatment, and environmental conditions for growth. Cell counting, protein assessment, or certain metabolic markers have been proposed as measures of cell quantity (León et al. 2013). Cell passage demonstrates security in phenotype and functional characteristics until passage no. 30 (Taciak et al. 2018). It is important to note that any change in the medium formulation can lead to variations and show differences in consumption rate, is recommended the use of formulations, suppliers, serum, additives and batches to ensure culture conditions standardization (Pamies et al. 2022). Once treatment or stimuli has been achieved, cells are washed and detached. Ice-cold or room temperature phosphate buffered saline (PBS), saline solution or water are employed to eliminate residual culture medium (between 1 or 4 washes) (Wang et al. 2020; Liu et al. 2017; Qu et al. 2025).

It has been reported that the detachment method used in cell culture can affect the intracellular extraction. Trypsinization has been shown to be an unsuitable method for physiological metabolomics since trypsin severely alters the cellular physiological state due to its interaction with membrane proteins, leading to sustained membrane damage in combination with metabolite leakage, all of which significantly alter metabolite levels and cellular metabolomic profiles (Bordag et al. 2016). Additionally, during the centrifugation, a significant portion of metabolites can be drained along with trypsin and washing buffers. Therefore, optimized extraction methods are being explored.

The use of scraping spatula as a detaching method has been proposed (Abdul-Hamid et al. 2019a, b; Bordag et al. 2016). At this step, the supernatant is removed, and cells can be stored at −80 °C if cell extraction is to be carried out later (Abdul-Hamid et al. 2019a, b). However, continuing the extraction step, the employment of scraping spatula and the use of the appropriate buffers, solvent concentrations, result in minor metabolome modifications (Teng et al. 2009; Martano et al. 2015; Ritter et al. 2008).

Intracellular samples for metabolomics analysis are extracted using organic solvents. Cold methanol (MeOH), for its high cellular permeability, is the most frequently used solvent. Rarely, hydrochloric acid (HCl), phosphoric acid (H_3_PO_4_), and perchloric acid (HClO₄) are used. Other options include a mixture of ethanol (EtOH)/water 75:25 (Muthubharathi 2021), or MeOH/water 80:20 suspended in ice-cold (Prechilled) (Abdul-Hamid et al. 2019a, b; Huang et al. 2023; Fan et al. 2022), acetonitrile/water 1:1 (Wang et al. 2020), or MeOH/trichloromethane/water 2.85:4:4 (Mei et al. 2019), often followed by immersion in liquid nitrogen process, sonication on ice (Li et al. 2025) vortex and centrifugation at 12,000 rpm or 120 g with times under 15 min at 4 °C (Abdul-Hamid et al. 2019a, b; Wang et al. 2020; Fan et al. 2022). Recently, the design of a simple, rapid, semi-automated method for preparing metabolomic samples from 20 μL of RAW 264.7 cells suspended in culture media has been reported. This approach employs filter-assisted electroporation for cellular synthesis and chilled organic solvent extraction to produce metabolomic samples from suspended cells within two minutes (Coulton and Edwards 2021).

Before using advanced chemical instruments for analysis, supernatant samples are subjected to an evaporation process with stream of nitrogen (Li et al. 2025; Fan et al. 2022; Wang et al. 2024), freeze/vacuum-dried (Huang et al. 2023), rotary evaporation (Wang et al. 2024) or kept in their solvent form (Lau et al. 2021; Li et al. 2022a, b). Different methods recommend storing the samples at −80 °C if instrumental analysis is not to be performed right away (Wang et al. 2025a, b; Palomares et al. 2023).

Different procedures have been described for sample preparation (Table 1). This step will vary depending on the type of analysis to be performed and the extraction method used. Specifically, samples that have been lyophilized or from which the solvent has been removed must be resuspended. Samples subjected to LC/MS–MS are dissolved in LC–MS-grade water or 0.1% formic acid (Huang et al. 2023; Qu et al. 2025), HPLC-Orbitrap-MS/MS or HPLC-QTRAP dissolved in 70% acetonitrile (Li et al. 2025; Wang et al. 2024), for ultra-high performance liquid chromatography (UHPLC) is used acetonitrile/water (Wang, et al. 2025a, b) and in analysis by UHPLC coupled with electrospray ionization (UHPLC/ESI-Q-Orbitrap) samples are dissolved with MeOH and filtered (Fan et al. 2022). For UHPLC/Q-Orbitrap high-resolution mass spectrometry (UHPLC/Q-Orbitra HRMS), samples without lyophilization process and extracted with prechilled MeOH are employed directly (Li et al. 2022a, b). Sample preparation for ^1^H NMR spectroscopy consists of redissolved sample in deuterium oxide (D_2_O) with PBS and trimethylsilylpropionic acid (TSP) or 3-(trimethylsilyl)-propanesulfonic acid sodium salt (DSS) (Song et al. 2022; Li et al. 2021a; Zhao et al. 2022; Palomares et al. 2023).

**Table 1:** Methods reported for intracellular content extraction

Cell density	Rinse step	Solvent	Extraction	Sample preparation for analytical instrument analysis	Analytical method	Reference
1 × 10^5^	PBS × 1	0.3 mL of prechilled 80% methanol	Well vortex, whirled, sonicated, centrifuged and freeze-dried	LC–MS-grade water	UHPLC-MS/MS	(Huang et al. 2023)
6 × 10^5^	Not specified	1 mL of precooled 80% methanol	Flash-frozen in liquid nitrogen, sonicated on ice, vortexed, centrifuged and dried with nitrogen	70% acetonitrile	HPLC-Orbitrap-MS/MS	(Li et al. 2025)
1 × 10^6^	PBS × 3	0.4 mL of 80% methanol at −80 °C	Homogenized, ice water bath, centrifuged and dried with nitrogen	1 mL of methanol and filtered	UHPLC/ESI-Q-Orbitrap	(Fan et al. 2022)
Not specified	Cold ultrapure water × 1	0.2 mL of pre-chilled extraction solvent (50% acetonitrile with 50 ng/mL FMOC-glycine)	Sonicated	Filtered	LC–MS/MS	(Lau et al. 2021)
Not specified	Not specified	Prechilled methanol	Not specified	Not specified	UHPLC/Q-Orbitrap HRMS	(Li et al. 2022a, b)
1 × 10^5^	PBS × 4	Cold 80% methanol containing undecanoic acid, nonadecanoic acid, and l-glutamic acid-d3	Vortexed, freeze-thawed with nitrogen, centrifuged, dried with rotary evaporation	70% acetonitrile	HPLC-QTRAP	(Wang et al. 2024)
Culture dishes (10 cm) at 80% of confluence	Precooled normal Saline solution	2 mL of precooled 80% methanol	Scrapped, crushed by ultrasonic crusher, centrifuged and dried with nitrogen	0.06 mL of 0.1% formic acid	LC–MS/MS	(Qu et al. 2025)
1 × 10^6^	PBS × 2	1 mL of methanol/acetonitrile/water	Cell scrapped, vortexed, low temperature sonication, incubation at −20 °C, centrifuged and dried under vacuum	0.1 mL acetonitrile/water (1∶1, v/v)	UHPLC	(Wang et al. 2025a, b)
Not specified	PBS × 3	1 mL ice-cold methanol/water solution (4:1, v/v)	Liquid nitrogen, incubation at −80 °C, vortexed, centrifuged and vacuum dried	0.1% formic acid–water and 0.1% formic acid–acetonitrile	UHPLC/Q-Orbitrap	(Ye et al. 2021)
Not specified	PBS × 3	4 mL of ice-cold methanol and methanol/trichloromethane/water (4/4/2.85)	Cell scrapped, vortexed, ultrasonicated and centrifuged and water evaporated	0.9 mL buffer (pH 7.4), centrifuged and lyophilized. 0.7 mL of _D2O_ and 0.05 mL of PBS prepared using D_2_O (pH 7.4) containing 0.1% TSP	^1^H NMR	(Song et al. 2022)
Not specified	PBS × 3	4 mL of ice-cold methanol and methanol/chloroform/ultrapure water (4/4/2.85)	Cell scrapped, vortexed, ultrasonicated and centrifuged and water evaporated	Dissolved in D_2_O, lyophilized and resuspended in 0.65 mL PBS with D_2_O (pH 7.4, containing 0.1% TSP)		(Li 2021a, b)
2 × 10^6^	2 mL of Hank´s solution and PBS × 2	1 mL of cold methanol/water (v/v)	Scrapped, ultrasonicated, centrifuged,	0.5 mL of D_2_O and DSS		(Palomares et al. 2023)
1 × 10^7^	PBS × 1	Acetonitrile/water (v/v)	Ultrasonicated, acetonitrile removed with nitrogen	99.8% D_2_O and PBS, containing 0.05% (w/v) of TSP		(Zhao et al. 2022)

Abbreviations: *PBS*, phosphate buffered saline; 1H−NMR, nuclear magnetic resonance; UHPLC−QTRAP, high purity liquid chromatography quadrupole−trap; LC-MS, liquid chromatography coupled with mass spectroscopy; UHPLC, ultra−high performance liquid chromatography; UHPLC/ESI−Q−Orbitrap coupled with electrospray ionization; UHPLC−Q−Orbitrap HRMS ultrahigh−performance liquid chromatography coupled to quadrupole Orbitrap high−resolution mass spectrometry; D_2_O, deuterium oxide; TSP, trimethylsilylpropionic acid; *DSS*, 3−(trimethylsilyl)−propanesulfonic acid sodium salt

Currently, multi-omics methods that allow the detection of a higher number of metabolites using small amount of sample material are being developed. Additionally, metabolomics research using a single cell has been achieved (Lanekoff et al. 2022; Seydel 2021). In response to the growing demand for fast and accurate measurement, new advances in metabolomics are being seen with an increasing focus on automation. Certain analytical methods offer advantages over others when considering factors such as sensitivity, reproducibility, resolution, sample volume for analysis, and simplicity of data interpretation, where tools like GC–MS and HPLC–MS offer higher sensitivity and resolution compared to NMR; Nevertheless, NMR provides enhanced reproducibility with minimal sample volume, it is a nondestructive analysis, along with the advantage that metabolite identification does not need specialized libraries (Munjal et al. 2022; Fraga-Corral et al. 2022).

Metabolite extraction standardization itself guarantees the strength, repeatability and reliance of acquiring intracellular components. There is no single method, since each type of sample requires specific sampling, collection, cooling and extraction. Before employing any new extraction method or analytical technique, and when handling new biological materials, it is essential to conduct thorough pilot experiments to fully assess the technical variation needed to design a statistically valid experiment (Alseekh et al. 2021). These limitations relate to the analytical procedures and the study framework (including consistency in sample collection and handling, technology, analysis, and data integration) and decrease the accuracy of the results. In fact, due to the significant variability of metabolites (chemical structure, physicochemical characteristics, and concentration), the process invariably exhibits some degree of bias, resulting from their extraction and detection methods (Monteiro et al. 2013; Pinu et al. 2019).

Biological replication is crucial, so, at least four replicates should be performed, although more replicates are suggested; the required number of replicates varies depending on the desired statistical power, effect size, and true variance (Alseekh et al. 2021). Information from standard quality controls evaluated across all studies and/or reference assay data from a selection of samples in each study can be utilized for normalization. Statistical models used to obtain ideal scale factors for each sample using the complete dataset, including normalization by unit norm or median intensity, or the maximum likelihood technique derived from the method created are also employed. Normalization using one or more internal standard compounds based on empirical guidelines such as specific regions, is the most common process (Sysi-Aho et al. 2007). Once the sample has been obtained, further analysis requires computational tools to visualize, characterize, quantify and understand the impact of the intracellular metabolite changes.

## Metabolite identification

Metabolomics aims to provide comparative quantitative assessments of metabolite levels across samples. These studies offer several benefits, such as direct readout of biochemical activity and alterations in phenotype (Sindelar and Patti 2020) by discrimination of increment/decrement in metabolite concentrations that led to modifications in inflammation-driven metabolites (Fitzpatrick and Young 2013) or metabolic pathways. Usually, these modifications are correlated with energetic processes and amino acids metabolism, as well as pathways related to the urea cycle, stress, antioxidant, creatine administration, and glutamine catabolism (Mei et al. 2019).

Various biomolecules amino acids, carbohydrates, and lipids are analyzed and characterized using advanced metabolomics approaches. NMR spectroscopy and mass spectrometry are the two most common techniques to collect metabolite profiles. The main advantages of these methods include their non-invasive approach, consistent results, and simple sample preparation as described previously (Tounta et al. 2021).

Many online databases are used to identify the metabolites in the samples. For example, the Human Metabolome Database (HMDB) was first released in 2007 and provides quantitative metabolomics services, offering complete coverage of the human metabolome with GC, MS/MS, and NMR spectra (Wishart et al. 2022). The Biological Magnetic Resonance Data Bank (BMRB) developed and pioneered by John Markley and Eldon Ulrich 40 years ago, has become an essential tool for structural biology in NMR. The BMRB repository has accumulated more than 14,000 curated entries (Baskaran et al. 2022).

CHENOMX is an NMR analysis software that works with an extensive spectral reference library to identify and quantify the concentration of visible compounds in NMR spectra, facilitating concentration measurement, identification, and advanced spectral analysis (Vitols and Colin 2006). BiGG (a knowledge base of Biochemically, Genetically, and Genomically structured genome-scale metabolic network reconstructions) provides information about a wide range of metabolites and their associated genes (Schellenberger et al. 2010). The Bayesian Automated Metabolite Analyzer for NMR Spectra (BATMAN) is an open-source R package designed to estimate metabolite concentrations from NMR spectral data. It separates peaks from one-dimensional NMR spectra, automatically correlates them to specific metabolites from a target list, and provides concentration estimates. It includes details about the typical peak patterns of metabolites and can consider variations in peak positions frequently seen in the NMR spectra of biological samples (Hao et al. 2014).

Automated spectraL processing system for NMR (AlpsNMR) is another R-package that provides automated signal. Signature mapping (SigMa), developed as a standalone tool using MATLAB dependencies, for processing raw ^1^H-NMR spectra into a metabolite table. NMR filter, a stand-alone interactive software for high confidence NMR compound identification that runs NMR chemical shift predictions. MSHub/Electron Ionisation (EI)-Global Natural Product Social (GNPS) Molecular Networking analysis, enables users to store, process, share, annotate, compare and perform molecular networking of both unit/low resolution and GC–HRMS data (Misra 2021).

**Fig. 3:**
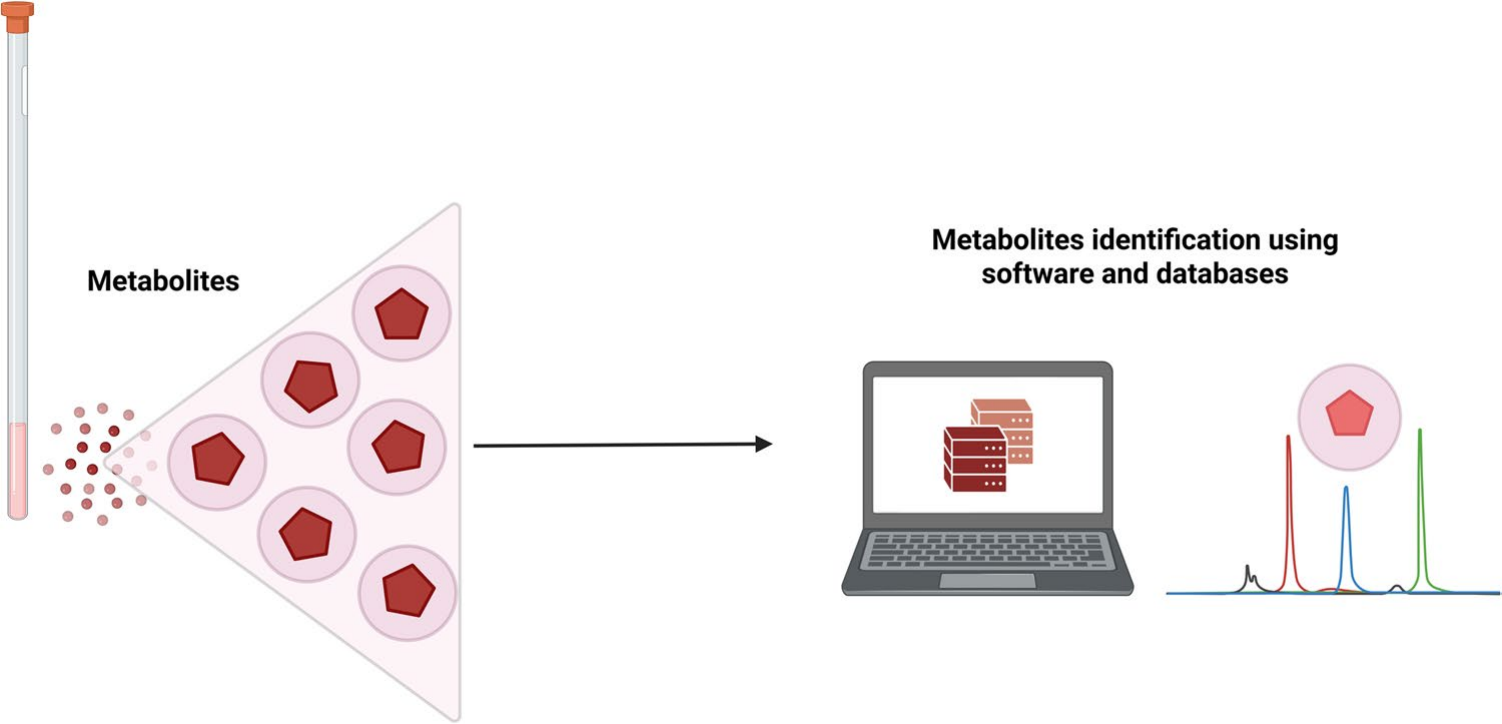
Metabolites identified by databases and software, ready to interpret them through Multivariate Analysis. Created in https://BioRender.com

Several other online databases used in metabolomic studies are the *E. coli* Metabolome Database (ECMDB), the Human Microbial Metabolome Database (MiMEDB) (Wishart et al. 2023), SetupX, BinBase, MassBank, METLIN, Golm Metabolome Database, Madison Metabolomics Consortium (MMC) Database (Fig. 3) (Rath et al. 2024).

Most programs and databases allow the extraction of structural signals using normalized data compared with standards, emulation or data obtained from other studies where the metabolite has been clearly validated. Results are usually presented in numerical format and can be examined statistically using multivariate analysis techniques.

## Multivariate analysis

For many years, multivariate analysis has been used in various fields, including informatics, engineering, and pharmacy, among others, to reduce a set of data of interest (Li et al. 2022a, b). It has been employed in analyzing taxonomic connections and classification of animal species, whereas in pharmacology and chemistry, it has played a role in the development and analysis of both new and existing drugs. These techniques are often applied using chemical data, a process called “chemometrics”, which allows the transformation of large amounts of chemical data through mathematics and statistics, used as a tool-linker of interactions between molecules and their biological activities. Therefore, chemometric analysis are used in metabolomic studies (Cornejo-Báez et al. 2020).

The role of analytical chemistry in providing simple and cost-effective solutions for key areas, specifically clinical, pharmaceutical, environmental, and agri-food, consistently faces challenges. Applying techniques to understand the cumulative impact of multiple species, assess the relationship between experimental factors on signal output, and differentiate or classify multiple targets simultaneously offers a valuable opportunity to move toward a multivariate perspective (Sun et al. 2025; Krzanowski and Wojtek 2014).

The primary purpose of this discipline is to extract meaningful chemical information from the obtained chemical data. Different chemometric techniques have unique characteristics, enabling the resolution of critical problems in chemistry due to the complex relationships in data matrix. Two principal approaches to the application of multivariate analysis can be distinguished, for classification and discrimination (Unsupervised and supervised methods) and for regression and prediction (Linear and Non-linear methods). Unsupervised methods such as Principal Component Analysis (PCA) explore variance within an X matrix. PCA converts matrix data consisting of multiple measured variables. To represent the data effectively, the primary axis is aligned with the matrix, thereby reducing the distance between data points and the axis (Konishi 2015). One of the advantages of this method is that the intrinsic data structure is preserved, whereas alternative approaches may distort the structure through the application of specific estimations (Konishi et al. 2019).

The second approach corresponds to supervised techniques such as partial least squares (PLS) and orthogonal partial least squares (OPLS), which examine the variability in X matrix that enables the prediction of Y response matrix (Tortorella and Cinti 2021). PLS is based on principal components regression, which allows the development of models capable of predicting multiple dependent variables. This analysis is used when the number of variables exceeds the number of composite variables within the dataset and when the selected variables are interrelated (Peter et al. 2019). Moreover, this approach enables the simultaneous relationship of several independent variables with large numbers of dependent variables (Popovic et al. 2019).

**Fig. 4:**
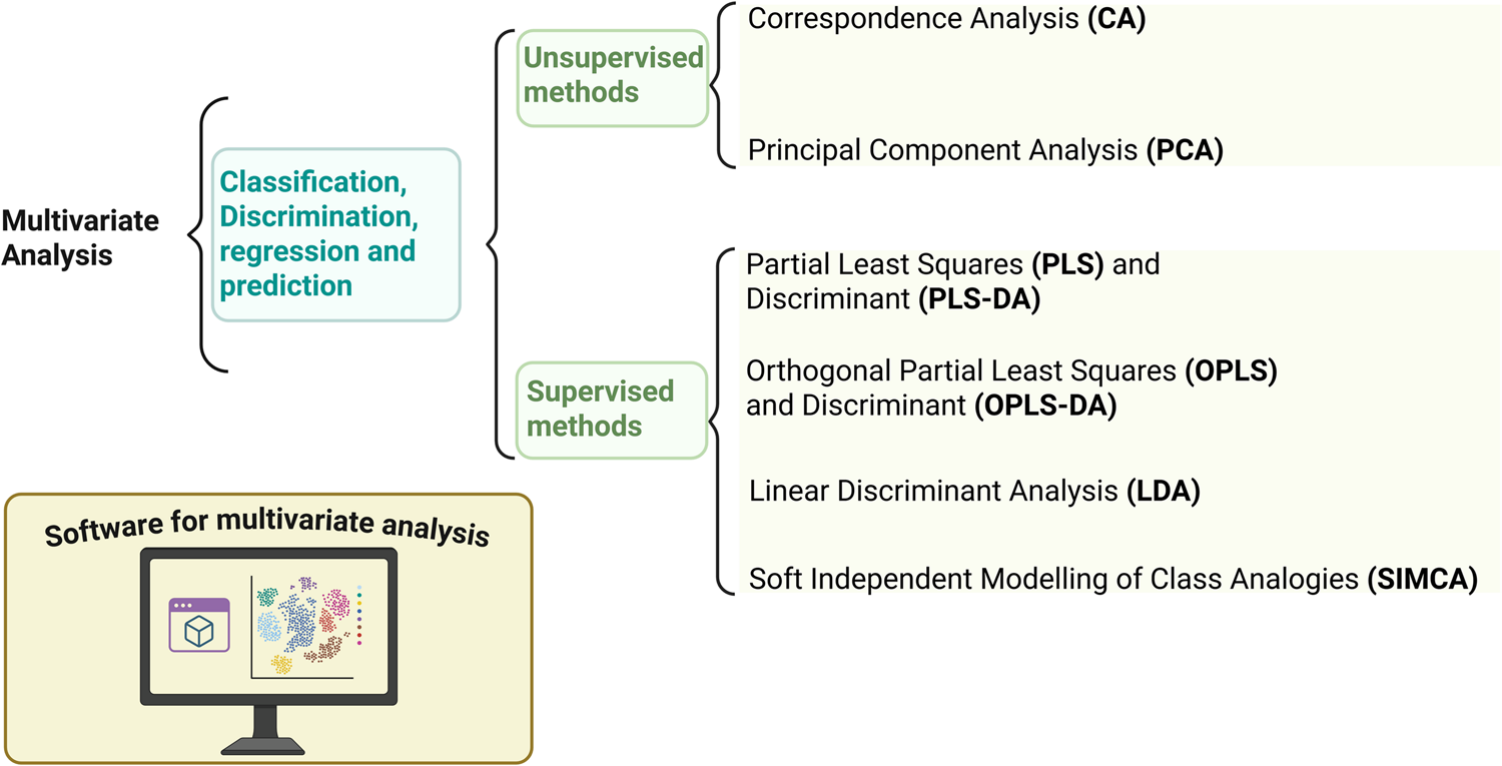
Multivariate Analysis classification, supervised and unsupervised methods are part of the classification and discrimination analysis. Adapted from (González-Domínguez et al. 2022). Created in https://BioRender.com

OPLS facilitates understanding by partitioning the variation of explanatory variables into two segments: "predictive" variation associated with the response, and “orthogonal” variation, which is variation not associated with the response. Both OPLS and PLS are equivalent for single-response variable models; however, OPLS has been recognized in fields where understanding the relationship between descriptive variables and the response is critical (Fig. 4) (Forsgren et al. 2025).

OPLS, OSC (Orthogonal signal corrected)-PLS and PLS as discriminant analysis (DA) are carried out to emphasize the distinction between sets of observations, thus achieving optimal separation between classes (Xu et al. 2024). OPLS-DA and PLS-DA remain extensively used in omics for analysis interpretation of biological variations among various classes. Applications involve distinguishing dietary intervention studies in metabolomics, patterns of metabolite distribution and selection of gene features using microarray data.

The existing market and its demand for tools that accelerate data analysis through multivariate analysis have benefited the release of proprietary software; SPSS, recognized for its intuitive menu-driven interface, provides a wide range of functionalities for different multivariate methods, such as MANOVA, factor analysis, and multiple regression (Sarker et al. 2024). STATA provides a wide range of functionalities for multivariate analysis, such as multiple regression, MANOVA, and multivariate time series analysis, all through an easy-to-use, command-based workflow (Mehmetoglu et al. 2021). SIMCA employs robust multivariate methods to uncover hidden patterns, trends, and relationships in large data sets into visual and understandable information (Anwardeen et al. 2023). R-studio and Python are open source software with a wide range of packages suitable for almost any type of multivariate analysis (Zelterman 2015; Kabir and Ahmed 2024). They are applied to develop models and condense them into clear results as plots.

Graphical representation for visualizing metabolomics data includes many types, but the most used are score plots, that display the scores of the first principal component against the scores of the second principal component; loading plots, that show the coefficients of each variable for the first component versus the coefficients of the second component. Scores indicate what is occurring with the samples, while loadings explain why it is happening in relation to the variables (Bevilacqua and Bro 2020). Finally, biplot overlays score and loading plot allowing concurrent interpretation of sample connections and the impacts and correlations of variables (Park et al. 2019). Multivariate analysis of intracellular metabolites of RAW 264.7 cells subjected to different treatments/stimuli will provide information to understand the role of metabolite variations in inflammatory processes. 

## Key metabolites and metabolic pathways related to inflammation in RAW 264.7 cells

### Energy production pathways

Most adenosine triphosphate (ATP) synthesis occurs during cellular respiration within the mitochondrial matrix, generating approximately 32 ATP molecules per oxidized glucose molecule. ATP provides energy essential for a variety of physiological processes, including ion transport, muscle contraction, nerve signaling, phosphorylation, and chemical synthesis. These processes, among others, generate a significant demand for ATP (Dunn and Grider 2023). ATP may integrate into arginine biosynthesis and subsequently be metabolized into fumaric acid, potentially impacting the tricarboxylic acid cycle (TCA) (Wang et al. 2024; Abdul-Hamid et al. 2019a, b). Moreover, ATP levels decreased significantly in macrophages subjected to LPS stimulation (Carrola et al. 2020).

Differences can be found in situations of high metabolic energetic demand when the rate of ATP use overwhelms its production (Palomares et al. 2023). A decrement in energy demand reflects a remarkable decrease in glucose consumption. However, it has been reported that in some cases ATP levels do not increase when glucose was used for energy production after the activation of RAW 264.7 cells by LPS, suggesting that the energy derived from glucose was insufficient to supply the increased energy requirements (Wang et al. 2020; Zhu et al. 2015). The function of ATP extends beyond energy processes. P2 purinergic receptors are activated by extracellular ATP released from dead or dying macrophage cells and modulate physiological processes covering wound repair, tissue balance, neurodegenerative conditions, immune responses, and inflammation (Wang et al. 2021a, b; Lombardi et al. 2021).

Glucose also plays a crucial role in energy use, enters the cytoplasm via glucose transporter 1 (GLUT1) and is metabolized through the glycolytic and the TCA cycle pathway. Prominent increases suggest the downregulation of the glycolytic conversion of glucose to pyruvate (Carrola et al. 2020; Abdul-Hamid et al. 2019a, b). Conversely, glucose levels decreased after LPS activation of RAW 264.7 cells, indicating that substantial amounts of glucose were used for energy production (Wang et al. 2020; Freemerman et al. 2014). Glycolysis is essential for macrophage function. Overexpression of GLUT1, a key glucose transporter, has been shown to increase glucose uptake and metabolism, thereby altering the inflammatory response of macrophages by increasing the release of inflammatory mediators in LPS activation (Liu et al. 2021; Ye et al. 2022).

Pyruvate, the product of glycolytic activity, can enter to different metabolic pathways, including the production of alanine by the alanine transferase (ALT) reaction. Low alanine excretion rates indicate a preference for the aerobic metabolism of energy production, supporting the hypothesis of upregulation of the TCA cycle (Palomares et al. 2023). Evidence suggests that pyruvate dehydrogenase kinase 1 (PDK1) deletion decreases the production of M1 macrophage-associated inflammatory mediators IL-6, interleukine-12 (IL-12), IL-1β, and iNOS, increasing M2 macrophages population. Pyruvate metabolism affects macrophage polarization, mitochondrial dynamics and membranes formation (Kim et al. 2023). Macrophages subjected to mild hypoxia, the hypoxia inducible factor (HIF-1α-PDK1) axis induces glycolytic reprogramming by converting pyruvate to lactate. Conversely, downregulation of dehydrogenase kinase 2 (PDK2) enhances macrophage activation (Li et al. 2023). Hypoxia inducible factor (HIF-1α) is also stabilized by excessive succinate in cytosol (Huang et al. 2024).

Respiration and glycolysis, during which pyruvate could produce energy through its metabolism to lactate and alanine, resulting in a significant increase of both (Wang et al. 2020). However, anaerobic respiration is less efficient than aerobic respiration in generating energy, hence the energy demands of LPS-activated cells are not covered (Wang et al. 2020).

Coenzyme nicotinamide adenine dinucleotide (NAD +) modulates the activity of NAD + -dependent enzymes and governs metabolic reactions, NAD + is reduced to NADH whereas NADH can be oxidized back to NAD + during anaerobic lactic fermentation or during mitochondrial oxidative phosphorylation. It has been reported that variations in intracellular NAD + affect the functional characteristics of innate and adaptive immune cells, playing a role in the inflammatory phases in TNF-α transcription (Fang et al. 2023; Wang et al. 2020; Iqbal and Zaidi 2006; Al-Shabany et al. 2016, 2014).

Itaconate, a signaling metabolite produced by activated macrophages (Yuk et al. 2023) is an important bridge among metabolism, inflammation, oxidative stress, and the immune response (Dunn and Grider 2023). Their role as a succinate dehydrogenase (SDH) inhibitor, also known as complex II of the electron transport chain, catalyzes the oxidation of succinate to fumarate in LPS-activated macrophages and supports the metabolic reprogramming that drives into a proinflammatory phenotype. Itaconate functions as a nuclear factor erythroid 2-related (Nrf2) activator and cysteine modifier, and a regulator of the activating transcription factor (ATF3)/IκBζ axis, glycolysis, type I IFNs, and the leucine-rich family, pyrin-containing 3 (NLRP3) inflammasome has been described beyond its regular roles in antimicrobial defense (Peace and O’Neill 2022; Yu et al. 2019). Finally, increased levels of itaconate are noted in LPS-stimulated macrophages due to downregulation of isocitrate dehydrogenase (IDH), and inhibition of SDH (Table 2) (Carrola et al. 2020; Yu et al. 2019).

**Table 2:** Metabolites in RAW 264.7 involved in energetic process pathways

Metabolite	Influence in inflammation	Increment/Decrement	Analytical method	Multivariate analysis	Reference
ATP	Energy supply to macrophage functions/Purinergic receptors (Cell lysis, apoptosis, degranulation, or membrane pore formation)	Decremented (M0 *vs* treatment macrophages)	^1^H-NMR and HPLC-QTRAP	PCA, PLS-DA, OSC-PLS-DA and OPLS-DA	(Carrola et al. 2020; Wang et al. 2020, 2024; Palomares et al. 2023; Abdul-Hamid et al. 2019a, b)
		Decremented (M0 *vs* M1 macrophages)			
		Incremented (M0 *vs* M1 macrophages)			
GTP	Energy supply to macrophage functions/Purinergic receptors (Cell lysis, apoptosis, degranulation, or membrane pore formation)	Decremented (M0 *vs *M1 macrophages)	HPLC-QTRAP	PLS-DA and OPLS-DA	(Wang et al. 2024)
Itaconate	SDH inhibitor	Incremented (M0 macrophages *vs* treatment)	^1^H-NMR	PCA, PLS-DA and OSC-PLS-DA	(Carrola et al. 2020)
Succinate	Stabilize HIF-1α				(Carrola et al. 2020)
Glucose	Release of proinflammatory mediators	Incremented and decremented (M1 *vs* treatment and M0 *vs *M1 macrophages, respectively)			(Wang et al. 2020; Abdul-Hamid et al. 2019a, b; Carrola et al. 2020)
NAD +	TNF-α transcription	Decremented (M0 *vs* M1 macrophages and treatment *vs* M1 macrophages)	^1^H-NMR and HPLC-QTRAP	PCA, PLS-DA, OSC-PLS-DA and OPLS-DA	(Wang et al. 2020, 2024)
NADP +			^1^H-NMR	PCA and OSC-PLS-DA	(Wang et al. 2020)
AMP	Not reported		^1^H-NMR and HPLC-QTRAP	PCA, PLS-DA, OSC-PLS-DA and OPLS-DA	(Wang et al. 2020, 2024)
Histidine		Decremented (M0 *vs* M1 macrophages)		PCA, PLS-DS and OSC-PLS-DA	(Wang et al. 2020; Fan et al. 2022)
Lactate	Energy supply to macrophage functions	Incremented (M0 macrophages *vs *treatment) Decremented (M0 *vs* M1 macrophages and treatment *vs *M0 macrophages)		PLS-DA, OSC-PLS-DA and OPLS-DA	(Wang et al. 2020, 2024; Palomares et al. 2023)

Abbreviations: *ATP,*adenosine triphosphate; *GTP,* guanosine triphosphate; *NAD+*, nicotinamide adenine dinucleotide; *NADP+* nicotinamide adenine dinucleotide phosphate; *AMP* adenosine monophosphate; *1H−NMR*, 1H nuclear magnetic resonance; *QTRAP* high purity liquid chromatography quadrupole−trap; *PCA *principal analysis component; *PLS* partial least squares; *OPLS* orthogonal partial least squares; *OSC,* orthogonal signal correction; *DA,* discriminant analysis

### Antioxidant pathways

Spermidine, aspartate and proline are amino acids that play a crucial role in the differentiation of unstimulated cells. The TCA and urea cycles are linked through the aspartate-arginosuccinate shunt, resulting in a reduction of intracellular aspartate, amino acid that promotes IL-1β secretion in M1 macrophages and boosts the activation of HIF-1α and inflammasome, incrementing the levels of metabolites in aspartate metabolism, such as asparagine (Lau et al. 2021; Vettore et al. 2021; Liu et al. 2021; Liu et al. 2020; Wang et al. 2021a, b; Kieler et al. 2021).

The production of proline is reduced after LPS activation, arginine is redirected for NO synthesis and during inflammation, inflammatory cells (M2-polarized macrophages and T helper 2 (Th2) cells) secrete cytokines that stimulate myofibroblast activation, also contributing to immune evasion (Wynn 2008; Kay et al. 2023). A reduction in intracellular proline, a metabolite of ornithine dysregulation, has been observed in LPS-stimulated cells, significantly affecting multiple metabolic processes, resulting in hyperornithinemia and hyperammonemia (Lau et al. 2021; Li et al. 2021a, b; Abdul-Hamid et al. 2019a, b). Betaine is recognized for its role as an osmoprotectant, methyl group donor in various physiological processes such as NF-κB and NLRP3 inflammasome inhibitor, there is evidence that it exhibits anti-inflammatory properties in various diseases (Zhao et al. 2018; Abdul-Hamid et al. 2019a, b).

Additionally, glutamine is the most abundant amino acid in blood with antioxidant characteristics and is proposed to be important for signaling by rapamycin target complex 1 (TOR1C) activation in LPS-induced M1 macrophages and inhibits HIF-1α by promoting the activity of HIF prolyl hydroxylases (Jiang et al. 2022). It is also the main glutamate provider to produce γ-l-glutamyl-l-cysteinyl-glycine known as glutathione (GSH). Glutamate is overproduced to counteract oxidative stress following LPS activation in macrophages (Wang et al. 2020; Viola et al. 2019). The decrement of glutamate suggests the participation of the cystine-glutamate antiporter system, which provides cysteine for the rate-limiting step of GSH synthesis and has been reported to be activated in macrophages following pro-inflammatory stimulation (Carrola et al. 2020; Wang et al. 2024; Abdul-Hamid et al. 2019a, b; Meiser et al. 2016).

The GSH redox system is the primary defense mechanism important for maintaining the redox balance and cellular membrane stability. LPS-activated RAW 264.7 macrophages show significantly decreased levels of glutathione, indicating enhanced antioxidant capacity (Wang et al. 2020; Huang et al. 2023). Phenylalanine metabolism also is closely related to the repair and antioxidant functions of cells, in inflammation inhibits the production of iL-1β and TNF-α in proinflammatory macrophages. Phenylalanine and valine can significantly increase the content of GSH and improve the activities of Cu/Zn superoxide dismutase (SOD1), catalase (CAT), and glutathione peroxidase (GSH-Px) by upregulating the mRNA expression of Nrf2 and downregulating the mRNA expression of Kelch-like ECH-associated protein 1 (Keap1) (Table 2). On the other hand, valine activates the PI3K/Akt1 pathway and promotes NO production (Fan et al. 2022; Huang et al. 2023; Zhang et al. 2023; Abdul-Hamid et al. 2019a, b; Chen et al. 2017).

Methionine and tryptophan metabolism are essential for macrophage activity and immune responses. Methionine is a vital amino acid that serves as a precursor to metabolites, such as s-adenosylmethionine (SAM), an important methyl donor in cellular functions (Huang et al. 2023). L-tryptophan and L-tyrosine are closely related to the repair and antioxidant function of cells and promote several key resolution processes, including the induction of pro-resolving proteins, such IL-10, and further enhancement of efferocytosis (Fan et al. 2022; Sukka et al. 2024). Taurine showed protective effects against oxidative stress and is remarkably decreased in the LPS group, participating in decreasing of TNF-α and IL-1β (Table 3) (Wang et al. 2020; Marcinkiewicz and Kontny 2014; Sartori et al.

2018).

**Table 3:** Metabolites in RAW 264.7 macrophages involved in antioxidant metabolic pathways

Metabolite	Influence in inflammation	Increment/Decrement	Analytical method	Multivariate analysis	Reference
Glutathione	Antioxidant capacity and upregulation of Nrf2	Decremented (treatment *vs* M1 macrophages)	^1^H-NMR and HPLC-QTRAP	PCA, PLS-DA, OSC-PLS-DA and OPLS-DA	(Wang et al. 2020; Huang et al. 2023; Abdul-Hamid et al. 2019a, b)
Taurine	Protection in oxidative stress and decrement of proinflammatory mediators	Decremented (M0 *vs* M1 macrophages)			(Wang et al. 2020, 2024)
Methionine	DNA methylation	Decremented (M0 *vs* treatment macrophages)	HPLC-QTRAP	PCA and PLS-DA	(Huang et al. 2023)
L-tyrosine	General antioxidant processes	Not specified			(Huang et al. 2023; Fan et al. 2022)
Proline	Activation of T lymphocytes and immune evasion	Incremented (M0 *vs* M1 macrophages)			(Lau et al. 2021; Huang et al. 2022; Abdul-Hamid et al. 2019a, b)
Phenylalanine	Increment of GSH content and influence in proinflammatory mediators	Decremented (M0 *vs* M1 macrophages), Incremented (treatment *vs* M1 macrophages)		PCA, PLS-DA, OSC-PLS-DA and OPLS-DA	(Wang et al. 2020; Huang et al. 2022; Fan et al. 2022; Abdul-Hamid et al. 2019a, b)
L-tryptophan	Influence on anti-inflammatory mediators and efferocytosis	Not specified		PCA, PLS-DA and OPLS-DA	(Huang et al. 2022; Fan et al. 2022)
Pantothenate	Attenuated antigen presentation and enhanced phagocytic activity	Decremented (treatment *vs* M0 macrophages)	^1^H-NMR and HPLC-QTRAP	PCA and PLS-DA	(Carrola et al. 2020; Fan et al. 2022)
Betaine	Influence on anti-inflammatory mediators, inflammasome inhibitor	Decremented (M0 *vs* M1 macrophages)		PCA, PLS-DA, OPLS-DA and OSC-PLS-DA	(Wang et al. 2020, 2024; Palomares et al. 2023; Abdul-Hamid et al. 2019a, b)
Ethanol	NLRP3 inflammasome and I influence on anti-inflammatory mediators	Decremented (treatment *vs* M0 macrophages)	^1^H-NMR	PCA and OSC-PLS-DA	(Wang et al. 2020)
Glutamine	Inhibits HIF-1α	Decremented (M0 *vs* M1 macrophages)	HPLC-QTRAP	PLS-DA and OPLS-DA	(Wang et al. 2024)
Valine	Promotes NO production	Incremented (M1 *vs* treatment macrophages)	^1^H-NMR and HPLC-QTRAP	PCA, PLS-DA, OPLS-DA and OSC-PLS-DA	(Wang et al. 2020; Huang et al. 2022; Fan et al. 2022)
Glutamate	Counteract oxidative stress	Decremented (treatment *vs* M0 macrophages and *vs* M1 macrophages)		PCA, PLS-DA, OSC-PLS-DA and PLS-DA	(Carrola et al. 2020; Wang et al. 2020, 2024)
β-Alanine	Fatty acids and pyruvate oxidation	Decremented (treatment *vs* M0 macrophages)	^1^H-NMR	PCA and PLS-DA	(Carrola et al. 2020)

Abbreviations: ^*1*^*H−NMR*, ^1^H nuclear magnetic resonance; *QTRAP,* high purity liquid chromatography quadrupole−trap; *PCA, *principal analysis component; PLS, partial least squares; OPLS, orthogonal partial least squares; OSC, orthogonal signal correction; *DA*, discriminant analysis

In addition, significant decreases were found for pantothenate and its precursor amino acids. Pantothenate are related with decreased expression of antigen presentation markers HLA-DRB5, HLA-DRB1 and HLA-DRA, suggesting that the high pantothenate synthesis and consumption exhibit attenuated antigen presentation and enhanced phagocytic function (Wang et al. 2025a, b). Its precursor amino acid β-alanine, possibly relates to the synthesis of coenzyme A, an essential factor in fatty acid synthesis and pyruvate oxidation decreased in M1 macrophages (Meiser et al. 2016). Finally, ethanol alleviates inflammation by lowering secretion of pro-inflammatory cytokines and inhibits NLRP3 inflammasome (Carrola et al. 2020; Xia et al. 2018; Nurmi et al. 2013).

### Amino acid metabolic pathways

The anaplerotic reactions are those that provide intermediates of the TCA cycle, decreased levels of aspartate promote IL-1β secretion in M1 macrophages boosting HIF-1α (Carrola et al. 2020; Fall et al. 2020). Alanine is produced in peripheral tissues by transamination of glutamate to pyruvate, significant decrease of alanine is consistent with changes in TCA cycle anaplerosis (Carrola et al. 2020; D'Souza et al. 2024).

Glycine levels may also be potentially related to the metabolic rewiring required to sustain the arginosuccinate shunt and/or creatine (Carrola et al. 2020), in inflammation glycine inhibits inflammatory responses and modulates the production of cytokines (Wu et al. 2020; Aguayo-Cerón et al. 2023). Glycine, as the product of purine metabolism, can enter the glycine, serine and threonine pathway. It can also be metabolized to produce betaine, whose role has been previously discussed (Wang et al. 2024). Dimethylamine markedly increased NO production, through increased expression of iNOS (Blackwell et al. 2011).

Decreased levels of threonine have been reported to trigger the complement system and immunoglobulins production, both are vital components of the non-specific humoral system (Carrola et al. 2020; Habte-Tsion et al. 2016). Changes in serine levels have been reported, this amino acid is required for both in vitro and in vivo, effector T cells proliferation and decrement Il-1β production and inflammasome activation trough target of rapamycin (mTOR) signaling (Wang et al. 2020; Carrola et al. 2020; Wu et al. 2020; Chen et al. 2020). Tyramine contributes to a proinflammatory state by an increment of TLR-4 (Patel et al. 2019).

Macrophages produce histamine involved in allergic diseases (Iwasaki et al. 2021). Spermidine also suppresses proinflammatory cytokines and improves the bioavailability of arginine required for NO biosynthesis (Madeo et al. 2018). 4-Hydroxyphenylpyruvate might be able to block both the inflammasome priming and activation processes and lead to a decrease in IL-1β production and pyroptosis in macrophages (Wei et al. 2022). Pyroglutamic acid is related to elevated IL-6 and serum amyloid A1 (SAA1) levels (Peris-Fernández et al. 2024).

A role for creatine in the regulation of macrophage polarization has been shown to be actively taken up by resting macrophages, being a potent endogenous inhibitor of pro-inflammatory IFN-γ-mediated, while supporting anti-inflammatory IL-4 induce polarization (Carrola et al. 2020). Incremented levels of creatine have been described in activated macrophages (Abdul-Hamid et al. 2019a, b; Wang et al. 2020). This leads to a switch from arginine to ornithine generation to produce NO and citrulline by expression of iNOS in arginine-creatine pathway (Lau et al. 2021). NO also leads to the inhibition of ornithine decarboxylase, which directly hinders the transformation of ornithine to putrescine to produce polyamines such as spermidine (Lau et al. 2021).

Lysine serves in crucial functions as substrate and regulator in numerous metabolic pathways (Wang et al. 2020). Acetate modulates inflammation through the G-protein and acetyl-CoA signaling pathways, reducing inflammation in mouse models of arthritis, colitis, and asthma (Table 4) (Moffett et al. 2020).

**Table 4:** Metabolites in RAW 264.7 macrophages in amino acid metabolic pathways

Metabolite	Influence in inflammation	Increment/Decrement	Analytical method	Multivariate analysis	Reference
Aspartate	Inflammatory mediators and HIF-1α	Decremented (M0 macrophages vs treatment) Incremented (M0 vs treatment and control macrophages)	^1^H-NMR and HPLC-QTRAP	PCA and PLS-DA	(Carrola et al. 2020; Lau et al. 2021)
Alanine	Not reported	Decremented (M0 macrophages vs treatment) Incremented (M0 vs M1 macrophages)	^1^H-NMR	PCA, PLS-DA and OSC-PLS-DA	(Carrola et al. 2020; Wang et al. 2020; Palomares et al. 2023)
Glycine	Anti-inflammatory mediators	Decremented (M0 macrophages vs treatment)	^1^H-NMR and HPLC-QTRAP	PCA, PLS-DA, OSC-PLS-DA and OPLS-DA	(Carrola et al. 2020; Wang et al. 2020, 2024)
Formate	Not reported	Decremented (M0 vs M1 macrophages)	^1^H-NMR	PCA and OSC-PLS-DA	(Wang et al. 2020)
Ornithine	NO production	Decremented (M0 vs M1 macrophages)	HPLC-QTRAP	PCA and PLS-DA	(Huang et al. 2023; Lau et al. 2021)
Citrulline		Incremented (M0 vs M1 macrophages)		PCA and OPLS-DA	(Lau et al. 2021; Huang et al. 2022)
Threonine	Not reported	Decremented (M0 vs treatment macrophages)	^1^H-NMR	PCA and PLS-DA	(Carrola et al. 2020)
Serine	mTOR signaling	Incremented (M0 vs treatment macrophages)		PCA, PLS-DA and OSC-PLS-DA	(Carrola et al. 2020; Wang et al. 2020)
Creatine	Inhibitor of IFN-γ-mediated	Incremented (M0 vs treatment macrophages) Decremented (M0 vs treatment macrophages)			(Carrola et al. 2020; Wang et al. 2020; Palomares et al. 2023; Abdul-Hamid et al. 2019a, b)
Leucine	Promote M2 polarization through mTORC1	No changes	^1^H-NMR and HPLC-QTRAP		(Wang et al. 2020)
Isoleucine	Not reported	Incremented (M0 vs M1 macrophages)	^1^H-NMR and HPLC-QTRAP	PCA, PLS-DA, OPLS-DA and OSC-PLS-DA	(Wang et al. 2020, 2024)
Lysine	Not reported	Decremented (M0 vs M1 macrophages)	^1^H-NMR	PCA and OSC-PLS-DA	(Wang et al. 2020)
Acetate	G-protein and acetyl-CoA signaling pathways	Decremented (M0 vs M1 macrophages)		PCA, PLS-DA and OSC-PLS-DA	(Wang et al. 2020; Palomares et al. 2023)
Homoserine	Not reported	Decremented (M0 vs M1 macrophages)	^1^H-NMR and HPLC-QTRAP	PLS-DA, OSC-PLS-DA and OPLS-DA	(Wang et al. 2020, 2024)
Pyroglutamate	Not reported	Decremented (M0 vs M1 macrophages)	^1^H-NMR	PCA and OSC-PLS-DA	(Wang et al. 2020)
Sarcosine	Activation of M2 macrophages	Decremented (M0 vs M1 macrophages)			
Dimethylamine	NO production	Incremented (M1 vs treatment macrophages)		PCA, PLS-DA and OSC-PLS-DA	(Wang et al. 2020; Palomares et al. 2023)
Tyramine	Regulation of TLR-4	Decremented (M0 vs M1 macrophages), incremented in treatment		PCA and OSC-PLS-DA	(Wang et al. 2020)
Histamine	Allergic processes	No changes			
Spermidine	NO biosynthesis	Decremented (M0 vs M1 macrophages)	HPLC-QTRAP	PCA	(Lau et al. 2021)
4-Hydroxyphenylpyruvate	Blocking inflammasome	Decremented (M0 vs M1 macrophages)		PLS-DA and OPLS-DA	(Wang et al. 2024)
Oxoglutaric acid	Not reported				
Pyroglutamic acid	Increment IL-6 and AA1				
2-Aminobutyrate	Not reported	Decremented (M0 vs M1 macrophages)	^1^H-NMR	PCA and PLS-DA	(Wang et al. 2020)
Aspartic acid	Not reported	Not specified		PCA and OPLS-DA	(Huang et al. 2022)

Abbreviations: ^*1*^*H−NMR*, ^1^H nuclear magnetic resonance; *QTRAP*, high purity liquid chromatography quadrupole−trap (QTRAP); *PCA,* principal analysis component; *PLS*, partial least squares; *OPLS*, orthogonal partial least squares; *OSC*, orthogonal signal correction; *DA,* discriminant analysis

### Phospholipid metabolism pathways

Notably, as an important component of biological membranes, phospholipids regulate membrane functions and provide substrates for the formation of bioactive molecules related to signal transduction, such as eicosanoids, phosphatidates, and diacylglycerols. Abnormal phospholipid metabolism induced by dietary factors is closely related to the development and progression of metabolic diseases (Ye et al. 2021).

Differential metabolites involved in pathways of biosynthesis of unsaturated fatty acids, insulin secretion, serine and threonine metabolism, linoleic acid metabolism, and inflammatory mediator regulation of Transient Receptor Potential (TRP) channels have been reported. Lipids are an energy source for macrophages, as during wound healing and tissue repair, fatty acid oxidation is the most important metabolic pathway of M2 macrophages (Huang et al. 2022). Elevated levels of phospholipids containing polyunsaturated fatty acids, can be easily oxidized to form oxidized phospholipids, controls the distribution of arachidonic acid and eicosanoid release in macrophages (Ye et al. 2021). Phosphatidylcholine inhibited the nuclear movement of essential transcription factors NF-κB, p65 and the AP-1 dimer subunits, c-Fos and c-Jun, leading to decreased production of inflammatory cytokines (Mizuno et al. 2025).

Marked reductions in the levels of numerous lysophospholipids along with increases in the levels of several phospholipids indicated changes in the function of lysophospholipid acyltransferases, phospholipases, and/or phospholipid transporters. Lysophospholipid acyltransferases and phospholipases control acylation and deacylation, thus influencing acyl chain composition and phospholipid ratio and subsequent changes in significant biological processes (Ye et al. 2021), lysophospholipids likely influence the function of classical mediators of vascular permeability, for example, histamine, serotonin, and bradykinins, also coordinate immune cells during inflammation (Kano et al. 2022). Elevated levels of lysophospholipids due to changes in membrane phospholipid metabolism are associated with impaired membrane structure and altered functions of membrane-associated enzymes, such as guanylate and adenylate cyclase (Sonkar et al. 2019). Phospholipids activate proinflammatory signaling pathways through Toll-like receptor (TLR)-mediated mechanisms (Raphael and Sordillo 2013). Cardiolipins are used by cells in mitophagy as an inefficient signal, triggering uncontrolled inflammation or cell death (Pizzuto and Pelegrin 2020). Monoolein exerts a suppressive impact on the generation of proinflammatory cytokines and blocks MAPK and NF-κB signaling pathways (Ali et al. 2017). Myo-inositol is a constituent of cell membranes, acting in cellular signaling and regulating enzyme activity and lipid transport. In inflammation and obesity has been shown that suppress TNF-α (Quarta et al. 2025). Significant decreases have been found for myo-inositol (Carrola et al. 2020; Abdul-Hamid et al. 2019a, b).

**Fig. 5:**
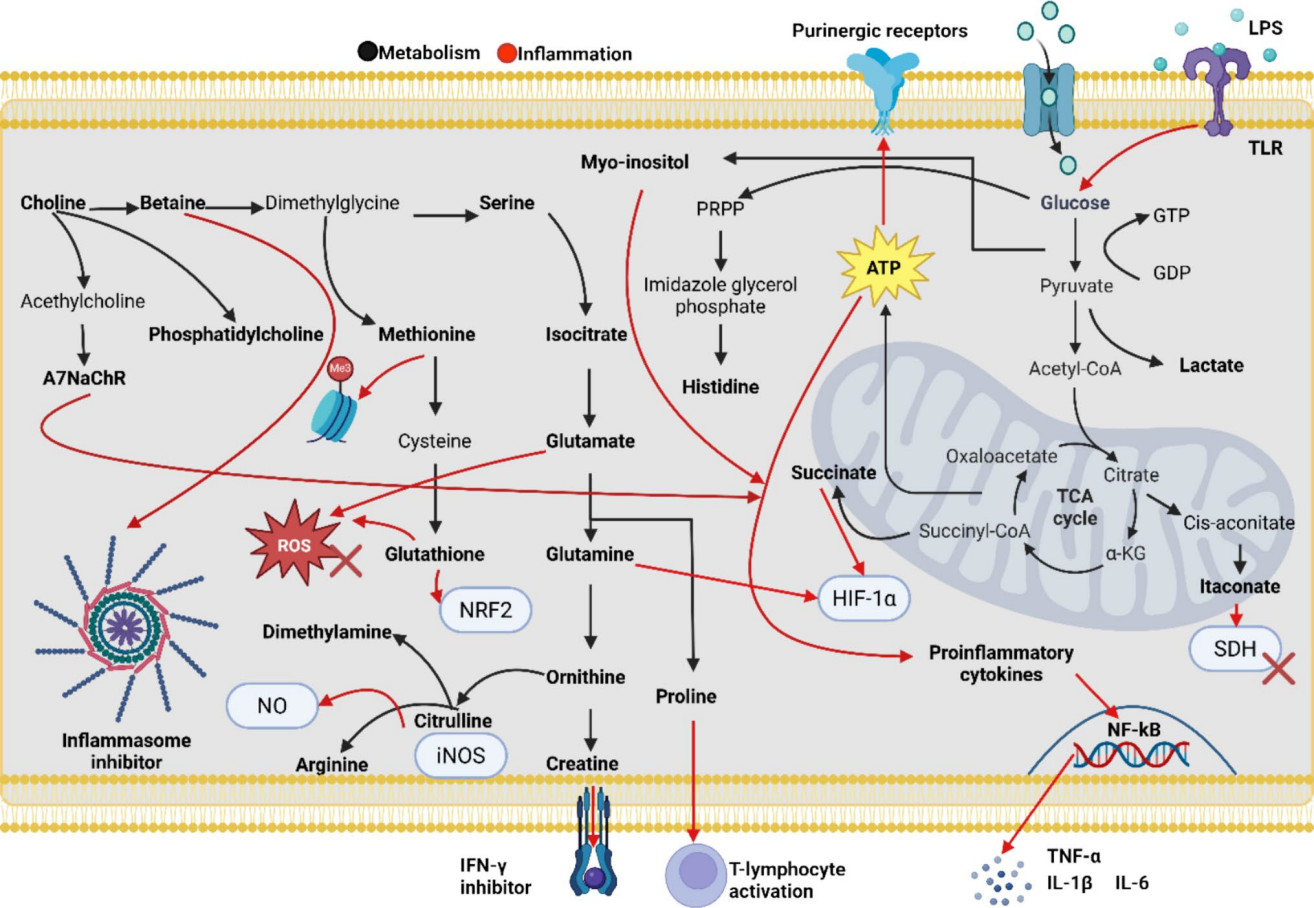
Inflammation and some metabolites interaction. Created in https://BioRender.com

The cholinergic pathway is stimulated to control the inflammatory response and minimize the inflammatory injury, thereby choline stimulates the synthesis and release of acetylcholine by reducing TNF-α, IL-1β, and IL-6 (Wang et al. 2020; Abdul-Hamid et al. 2019a, b). Trimethylamine N-oxide trigger vascular inflammation and endothelial dysfunction through the formation and activation of NLRP3 inflammasome in endothelial cells (Table 5) (Zhang et al. 2019). The interaction of various metabolites with the described inflammatory processes has been represented (Fig. 5).

**Table 5:** Metabolites in RAW 264.7 macrophages involved in phospholipid metabolism pathways

Metabolite	Influence in inflammation	Increment/decrement	Analytical method	Multivariate analysis	Reference
Lysophosphatidates	Innate and adaptative responses, host-defense, resolution processes	Decremented (M0 vs treatment macrophages)	HPLC-QTRAP	PCA	(Ye et al. 2021)
Lysophosphatidylcholines					
Lysophosphatidylethanolamines					
Lysophosphatidylglycerols					
Lysophosphatidylinositols					
Lysophosphatidylserines					
Phosphatidylethanolamines	Activate proinflammatory signaling pathways	Incremented (M0 vs treatment macrophages) Incremented (M0 vs treatment macrophages)			
Phosphatidylglycerol’s					
Phosphatidylinositol’s					
Phosphatidylserines					
Cardiolipins	Mitophagy				
Monoolein	Attenuation of MAPK and NFκB	Not specified		PCA and OPLS-DA	(Huang et al. 2022)
Elaidic acid	Not reported				
Eleostaric acid	Not reported				
Glycerophosphocholine	Influence inflammation parameters	Decremented (M0 vs M1 macrophages)		PLS-DA and OPLS-DA	(Wang et al. 2024)
Myo-inositol	TNF-α supression	Decremented (M0 macrophages vs treatment)	^1^H-NMR	PCA and PLS-DA	(Carrola et al. 2020; Abdul-Hamid et al. 2019a, b)
Sn-glycero-3-Phosphocholine	Not reported	Decremented (M0 vs treatment macrophages)		PLS-DA	(Palomares et al. 2023)
Trimethylamine N-oxide	Activation of inflammasome				
Choline	Cholinergic pathway	Incremented (M1 vs treatment; M0 vs treatment)	^1^H-NMR	PCA, PLS-DA and OSC-PLS-DA	(Wang et al. 2020; Palomares et al. 2023; Abdul-Hamid et al. 2019a, b; Ye et al. 2021)
Phosphatidylcholine	Influence on inflammation mediators	Not specified	HPLC-QTRAP	PCA and OPLS-DA	(Huang et al. 2022)
O-phosphocholine	Not reported	Decremented (M0 vs treatment macrophages)	^1^H-NMR	PLS-DA	(Palomares et al. 2023)

Abbreviations: ^1^H nuclear magnetic resonance (1H−NMR), high purity liquid chromatography quadrupole−trap (QTRAP), principal analysis component (PCA), partial least squares (PLS), orthogonal partial least squares (OPLS), orthogonal signal correction (OSC), discriminant analysis (DA)

The applications of this model are related to the use of several drug treatments, including natural products. Some examples include the use of extracts from unripe citrus fruits, which mitigate metabolic changes in LPS groups, alterations closely related to specific inflammatory processes (Li et al. 2025). On the other hand, the PAP1B protein derived from the areca nut indicates that the C-type lectin receptor signaling pathway is the most enriched in multivariate analysis (Ji et al. 2025). One study examined the exopolysaccharides of *Cyclocarya paliurus*, showing that their metabolic impact is associated with energy metabolism, protein/amino acid metabolism, lipid metabolism, nucleotide metabolism and other metabolic pathways, linking with inflammation in a similar way to that described above, and in the same way, the influence is categorized by energy metabolism, amino acid metabolism, lipid metabolism and nucleotide metabolism (Wang et al. 2025a, b).

## Conclusions

Cell lines as a metabolomic model investigate metabolic markers, contributing to understanding the processes occurring in several health conditions such as inflammation. Future efforts should focus on establishing the clinical relevance and utility of markers derived from metabolomics in cells and tissues, along with their successful implementation in large-scale clinical and pharmaceutical settings. Metabolomics faces several challenges, including the perception that scientists prioritize other omics fields, the cost of analytical equipment, and the financial burden of data processing. While numerous free software applications exist, premium options are often high-priced. The management of this data requires a diverse team with the expertise needed to effectively implement a metabolomic project. In addition to the obstacles in data collection, there are challenges in the identification and measurement of metabolites. In-house procedures result in differences between studies, leading to heterogeneous results even with the same model. Metabolites described in this review have been found in intracellular extractions of RAW 264.7 cell line challenged by an inflammation stimulus. We suggest that it is essential to bring together all the studies that have used immune cell lines and compare their results to those observed when using serum or tissues to establish a connection to animal models. Finally, metabolomics of RAW 264.7 intracellular content has shown to be a model to understanding the cellular mechanisms of inflammation and its stimuli; hence, it could be used for the identification of potential new treatments, reducing the cost and the time required to carry out the activity evaluation.

## Data Availability

All source data for this work (or generated in this study) are available upon reasonable request.
